# Mesenchymal stem cells therapy in companion animals: useful for immune-mediated diseases?

**DOI:** 10.1186/s12917-019-2087-2

**Published:** 2019-10-22

**Authors:** Inês Esteves Dias, Pedro Olivério Pinto, Luís Carlos Barros, Carlos Antunes Viegas, Isabel Ribeiro Dias, Pedro Pires Carvalho

**Affiliations:** 1 CIVG - Vasco da Gama Research Center, Vasco da Gama University School, Av. José R. Sousa Fernandes 197, Campus Universitário - Bloco B, Lordemão, 3020-210 Coimbra, Portugal; 2 Coimbra University Veterinary Hospital, Av. José R. Sousa Fernandes 197, 3020-210 Coimbra, Portugal; 3 Department of Veterinary Sciences, School of Agricultural and Veterinary Sciences, University of Trás-os-Montes e Alto Douro, Quinta de Prados, 5000-801 Vila Real, Portugal; 4 3B’s Research Group, I3Bs – Research Institute on Biomaterials, Biodegradables and Biomimetics, University of Minho, Headquarters of the European Institute of Excellence on Tissue Engineering and Regenerative Medicine, AvePark, Parque de Ciência e Tecnologia, Zona Industrial da Gandra, 4805-017 Barco, Guimarães Portugal; 5 ICVS/3B’s – PT Government Associate Laboratory, 4805-017 Braga/Guimarães, Portugal; 6 Vetherapy, 479 St, San Francisco, CA 94103 USA

**Keywords:** Mesenchymal stem cells, Immunomodulation, Canine atopic dermatitis, Feline chronic gingivostomatitis, Inflammatory bowel disease, Feline asthma

## Abstract

Mesenchymal stem cells are multipotent cells, with capacity for self-renewal and differentiation into tissues of mesodermal origin. These cells are possible therapeutic agents for autoimmune disorders, since they present remarkable immunomodulatory ability.

The increase of immune-mediated diseases in veterinary medicine has led to a growing interest in the research of these disorders and their medical treatment. Conventional immunomodulatory drug therapy such as glucocorticoids or other novel therapies such as cyclosporine or monoclonal antibodies are associated with numerous side effects that limit its long-term use, leading to the need for developing new therapeutic strategies that can be more effective and safe.

The aim of this review is to provide a critical overview about the therapeutic potential of these cells in the treatment of some autoimmune disorders (canine atopic dermatitis, feline chronic gingivostomatitis, inflammatory bowel disease and feline asthma) compared with their conventional treatment.

Mesenchymal stem cell-based therapy in autoimmune diseases has been showing that this approach can ameliorate clinical signs or even cause remission in most animals, with the exception of canine atopic dermatitis in which little to no improvement was observed.

Although mesenchymal stem cells present a promising future in the treatment of most of these disorders, the variability in the outcomes of some clinical trials has led to the current controversy among authors regarding their efficacy. Mesenchymal stem cell-based therapy is currently requiring a deeper and detailed analysis that allows its standardization and better adaptation to the intended therapeutic results, in order to overcome current limitations in future trials.

## Background

Regenerative medicine results from the need to treat diseases for which modern medicine has no accessible or effective treatment [1]. This kind of therapy relies on the use of cells as therapeutic agents capable of regenerating damaged organs and tissues [2, 3]. Despite the wide range of potential candidates that can be applied in these therapies, stem cells are the ones that have been continuously addressing greatest expectations within the scientific community [4].

In traditional textbooks, stem cells were believed to be at the origin of all main types of tissue and it was also stated that differentiation occurred unidirectionally and irreversibly, once they were totally or partially differentiated in a certain type of cell, they could not differentiate again. Currently, it is well known that these cells are characterized by their plasticity and can undergo transformation more than what was originally thought [5]. In fact, stem cells are now considered unspecialized cells that share two important characteristics, they can self-renew indefinitely or differentiate into more mature cells with specialized functions [1, 6].

**Fig. 1:**
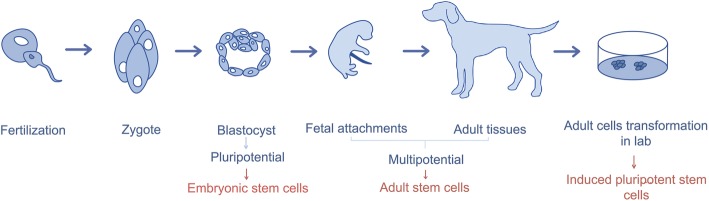
Stem cells differentiation

These cells can be classified in Embryonic Stem Cells (ESCs), Induced Pluripotent Stem Cells (iPSCs) and Mesenchymal Stem Cells (MSCs), which differ in origin, plasticity, differentiation potential and in risk of tumorigenesis. The process of differentiation of stem cells is represented in Fig. 1, and the classification of these cells in Table 1.

**Table 1:** Stem cells classification [1, 3, 4, 7, 8]

	ESC	iPSC	MSC
Origin	Derived from the inner cell mass of the pre-embryonic blastocyst	Derived from reprogrammed donor cells by exposure to transcription factors	Derived from tissues of endodermal, mesodermal and ectodermal lineages
Plasticity	Totipotents	Pluripotents	Multipotents
Differentiation potential	Endodermal, mesodermal and ectodermal tissues	Endodermal, mesodermal and ectodermal tissues	Mesodermal tissues
Risk of tumorigenesis	Yes	Yes	No (minimal)

ESCs are totipotent cells. They are able to originate any type of cells from the three germinal layers (endoderm, mesoderm, ectoderm) as well as extraembryonic annexes (e.g. placenta and umbilical cord). Despite their enormous potential, their application in cell therapy carries a series of inconveniences. Obtaining these embryonic cells is a much more complex and expensive procedure than other types of stem cells, there is a larger risk of tumorigenesis and immune rejection and there are also moral and legal restrictions to its use [4–6, 9, 10].

iPSCs are obtained from adult cells transformation which are modified in laboratory to pluripotent cells. Similarly to ESCs, iPSCs also present a high risk of tumorigenesis, limiting its therapeutic use [11, 12].

MSCs are multipotent cells (partially specialized cells capable of generating a certain number of cell types, presumably from its own original germinal layer) with self-renewal capacity [3, 6, 13, 14]. They derive from the embryonic layer of the mesoderm and under certain conditions they can differentiate into osteoblasts, chondrocytes, myocytes, β-pancreatic islets cells, neural cells, among others [15]. Adult MSCs have been shown to be nontumourigenic and nonimunogenic [4]. These cells were first successfully isolated from bone marrow by Friendstein and co-workers (1970) [16], and since then, the interest in their therapeutic potential has grown, leading to the emergence of new researches. These cells have been used in different veterinary hospitals as a therapeutic tool, however, there is yet no standard regulatory pathway for their use from the Food and Drug Administration (FDA) or the European Medicines Agency (EMA), since more data and definition of standardized protocols are required [6].

Currently, MSCs are mainly used in veterinary medicine to treat musculoskeletal system disorders, considering their ability to differentiate into various tissues of mesodermal origin. In human medicine, therapies with MSCs are mostly used in the treatment of immune-mediated inflammatory and ischemic diseases [17, 18]. Moreover, studies to evaluate the therapeutic potential of stem cells in humans would benefit from more companion animals models, since some diseases affect both alike, making them good preclinical models [11, 19].

The increase of immune-mediated diseases in veterinary medicine (such as canine atopic dermatitis, feline chronic gingivostomatitis, inflammatory bowel disease, feline asthma, among others) [20–22] has led to a growing interest in the research of these disorders and their medical treatment. Conventional immunomodulatory drug therapy such as glucocorticoids, or other novel therapies such as cyclosporine or monoclonal antibodies, are associated with numerous side effects that limit its long-term use [23], leading to the need for more effective and safe therapeutic strategies’ development.

The aim of this review is to provide a critical overview about the therapeutic potential, current status and future prospects of these cells in the treatment of immune-mediated diseases in veterinary medicine, pointing out the advantages and disadvantages of a treatment drawn with stem cells in comparison to other conventional treatments of these particular diseases.

## Mesenchymal stem cells

### Mesenchymal stem cells characterization

MSCs, also known as mesenchymal stromal cells, are multipotent cells, of non-hematopoietic origin, with self-renewal capacity, present in connective tissue throughout the body [14, 24]. They have been isolated from different adult tissues (such as bone marrow, adipose tissue, peripheral blood, muscle, dental pulp, periodontal ligament, articular cartilage, periosteum) and from extraembryonic tissues (for instance umbilical cord blood, membrane and amniotic fluid) [4].

In veterinary medicine, MSCs are obtained mainly from adipose tissue and bone marrow (BM), taking into consideration their location and harvesting procedure [3, 6, 25]. Although BM represents an abundant source of MSCs, harvesting this tissue is more invasive and requires younger donors, making adipose tissue a first choice [3, 10].

MSCs play an important role in the regulation of the immune system. Moreover, they are relatively easy to isolate and can be expanded in culture. Although MSCs are not part of the immunologic system according to the prearranged definitions [26], they interact with all kinds of immunologic cells. They produce a great variety of anti-inflammatory and pro-inflammatory factors, among which are cytokines, chemokines and prostaglandins, which target immune cells and affect their function [24]. It is noteworthy that MSCs are considered safe, with minimal or non-teratogenic risk and can be used for tissue repair and regeneration [14].

These cells present remarkable pleiotropic properties, including antiapoptosis, angiogenesis, growth factor production, antifibrosis and the ability to migrate toward injury sites through chemiotaxis [3, 6, 27]. The MSCs ability to differentiate into parenchymal cells of the mesoderm has become one of the main criteria to define their identity. Although recent data suggest that under appropriate culture conditions MSCs may also be induced to transdifferentiate into ectoderm (epithelia and neurons) and endoderm cells (lung cells and gut epithelial cells) [14, 27].

### Minimal criteria for defining mesenchymal stem cells

**Fig. 2:**
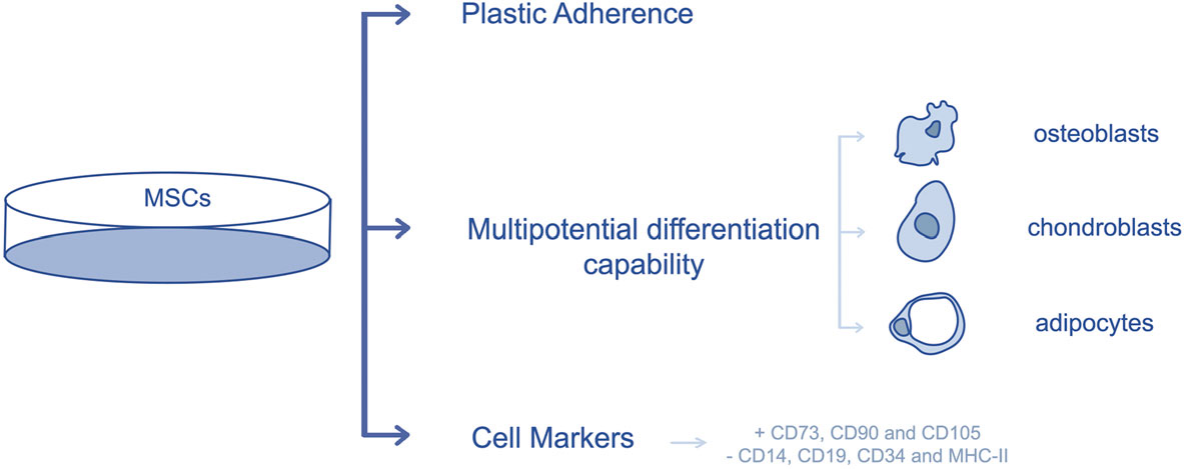
Characterization of mesenchymal stem cells

The pleiotropic nature of MSCs presents a challenge in their identification [27]. In order to create a broader consensus on the universal characterization of MSCs, and facilitate the exchange of data among investigators, the International Society for Cellular Therapy formulated minimal criteria for defining MSCs. These criteria include plastic adherence, capability for differentiation towards osteoblasts, adipocytes and chondroblasts under standard in vitro conditions, cell surface expression of CD73, CD90, CD105 and absence of hematopoietic stem cell markers (CD14, CD19, CD34, MHC-II, etc.) [4, 6, 28–30] (Fig. 2).

Although stem cell scientists continue to develop more stringent criteria, these basic criteria are generally accepted as the baseline for declaring a cell type as a MSC [5].

### Origin of mesenchymal stem cells

**Fig. 3:**
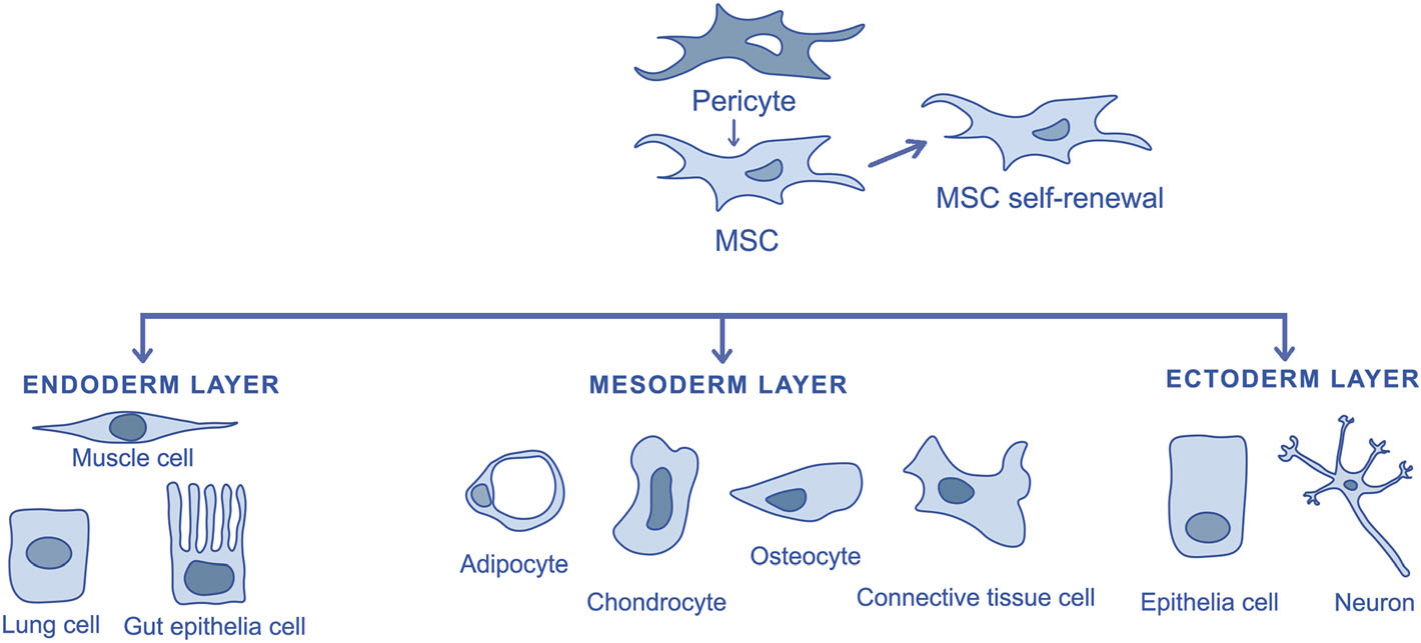
Possible origin of mesenchymal stem cells and their differentiation into mesodermal, endodermal and ectodermal cells

The origin of MSCs and their development is not yet fully understood [6, 31]. Although MSCs had formerly been isolated from bone marrow, they have already been withdrawn from the stroma of multiple organs and tissues including the adipose tissue, the tonsils, the umbilical cord, the skin and the dental pulp. Crisan and co-workers (2008) suggest as hypothesis that MSCs generate from pericytes (Fig. 3). Pericytes are perivascular cells present in the microvasculature of every vascularized connective tissue. This group has identified pericytes in multiple human organs (skeletal muscle, pancreas, adipose tissue and placenta) based on the expression of cell markers: CD146, NG2 and PDGF-Rβ. They found out that these cells expressed typical MSCs markers and in a specific culture medium they could differentiate into myocytes, osteocytes, chondrocytes and adipocytes [27, 32, 33].

Although the study has not directly monitored the probable in vivo transition of the pericytes to MSCs, they recognized pericytes as potential progenitor cells to non BMSCs [27, 32]. In 2011 Feng et al. provided evidence that MSCs originate from pericytes as well as non-pericytes. This group conducted an experiment using genetic lineage tracing. They suggest that the contribution of pericytes to the origin of MSCs depends on the extent of vascularity and tissue growth kinetics. Therefore, in tissues with low vascularization (e.g., articular cartilage), the contribution of the pericyte to MSCs will be lower than in tissues with more extensive blood supply [34]. Nevertheless, on the regenerative medicine field this hypothesis does not gather everyone’s consensus and acceptance.

### Interaction between mesenchymal stem cells and immune cells

MSCs have two important effects on the immune system, including an anti-inflammatory and immune-enhancing response. These cells regulate immune responses such as altering antibody production by B-lymphocytes, shifting T-lymphocyte subtypes, and inducing immune tolerance to allogeneic transplants [6, 35]. They release more than 200 bioregulatory products that have antimicrobial, immunomodulatory, antifibrotic, antiapoptotic, hematopoietic stem cells support, chemoattraction, angiogenesis, neuroprotective and mitogenic functions [4].

MSCs are capable of interacting with various types of immune cells, including T cells, B cells, natural killer (NK) cells, dendritic cells (DCs), macrophages/monocytes and neutrophils [36] affecting both the innate and humoral immune responses [35]. It is ambiguous whether MSCs should be classified as “immunosuppressive,” suggesting a nonspecific downregulation of the immune system, or rather if they induce an “immune tolerance,” suggesting a more specific suppression of aberrant immune responses. What seems to be clear is the fact that the MSCs’ immunomodulatory ability depends on several factors such as MSCs activation, MSCs tissue of origin, MSCs doses, MSCs time of administration, and MSCs contact with cells of the immune system [35].

**Fig. 4:**
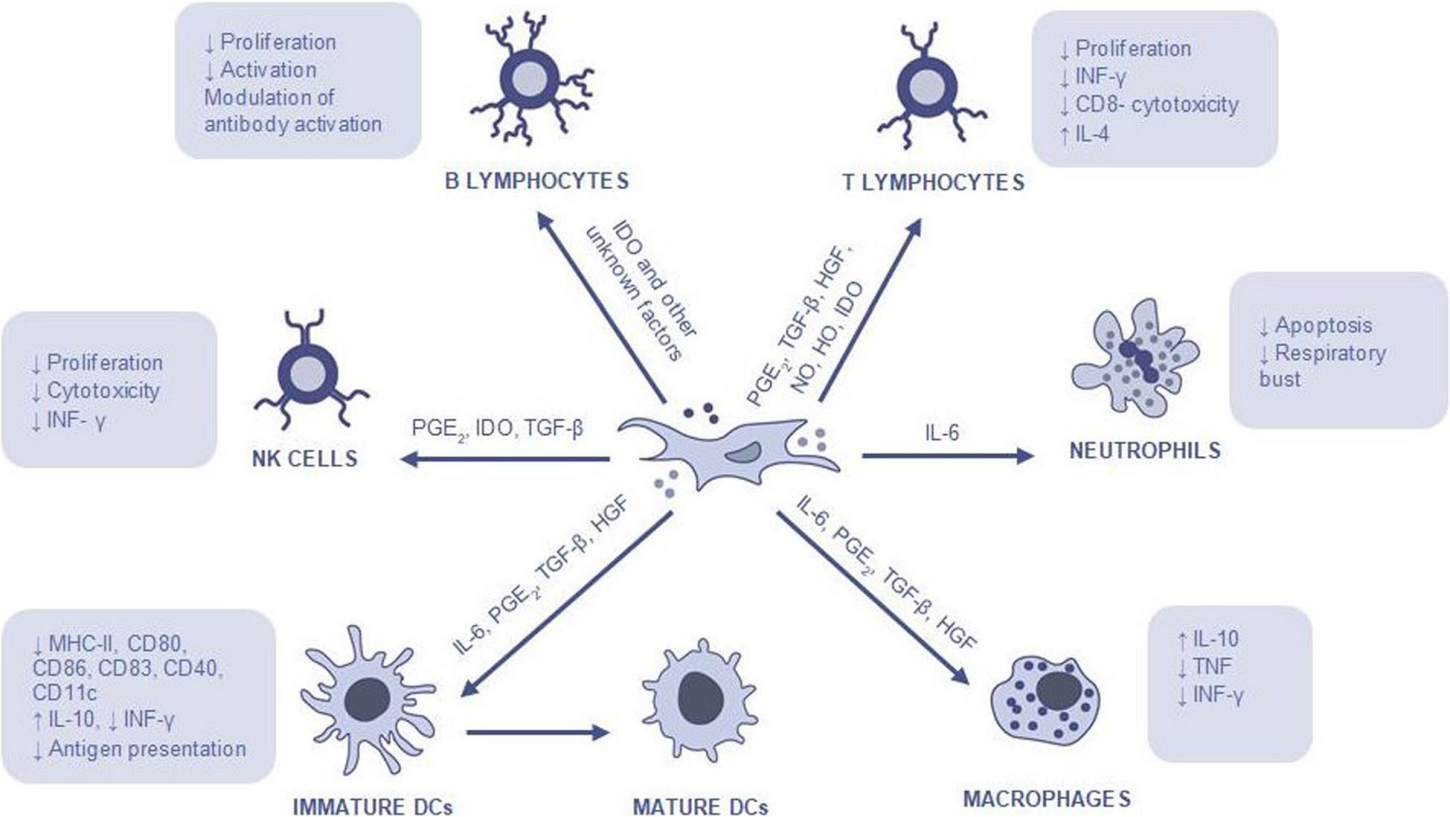
Mesenchymal stem cells’ mechanism of action and their interaction with immune cells

Although the underlying mechanisms of MSCs immunomodulation have yet to be elucidated, it is believed that immunomodulation first takes place through paracrine effects by producing immunomodulatory mediators, including nitric oxide (NO), indoleamine 2,3-dioxygenase (IDO) [37], transforming growth factor-β (TGF-β), hepatocyte growth factor (HGF), hemoxygenase (HO), interleukin (IL)-6 and prostaglandin E_2_ (PGE_2_), and they may also occur through cell-cell direct contact [6, 15, 38, 39] (Fig. 4).

### Mechanisms of mesenchymal stem cells suppression of innate immune cells

The innate immune cells play an important role in the organism homeostasis and are the first defence line against invading pathogens such as viruses and bacteria. The cells belonging to this system respond promptly in a non-specific form to pathogens [27]. This defence response is based on inflammation. During inflammation there are certain changes in the tissues caused by microbial invasion or tissue damage, resulting in the increase of blood flow and the local accumulation of cells, which may attack or destroy invaders. The innate immunity has a cellular defence line (neutrophils, monocytes/macrophages, DCs and NK cells) and an enzymatic defence line (these enzymes form what is known as the complement system) [40].

MSCs act on the immunity system through three main mechanisms. One of those mechanisms consists in activating proinflammatory monocytes and macrophages. In the presence of MSCs and their soluble factors (IL-6, PGE_2_, TGF-β, HGF), the M_1_ classic macrophages possessing proinflammatory functions, become active in M_2_ anti-inflammatory macrophages, which are characterized by high expression of IL-10, low production of tumour necrosis factor (TNF) and low production of interferon gamma (IFN-y) [38, 41, 42].

MSCs can also inhibit the differentiation of monocytes into mature DCs by releasing soluble factors such as IL-6, PGE_2_, TGF-β, HGF. These tolerogenic DCs produce high levels of IL-10 and have low capacity to stimulate proliferation of allogeneic T cells in a mixed lymphocyte reaction [38, 43].

Finally, MSCs inhibit NK cells proliferation and cytotoxicity, which can require cell-to-cell contact or can be mediated by soluble factors, including mainly PGE_2_ and IDO, but also TGF-β [14, 38, 44].

### Mechanisms of mesenchymal stem cells suppression of adaptive immune cells

The acquired immune system is responsible for recognizing foreign invaders, destroy them and retain the memory after first antigen contact. If the animal finds the same antigen a second time, the immunologic system will respond in a more effective and prompt way. The acquired immune system is based on two main branches. One is called “humoral immune response” and is mediated by B-lymphocytes that produce antibodies against exogenous invaders. The other major branch is called “cell-mediated immune response” and is mediated by T-lymphocytes which act directly against endogenous invaders that invade cells [40].

MSCs are able to suppress the proliferation of T cells and modulate their response through the secretion of several soluble factors (PGE_2_, TGF-β, HGF, NO, HO, IDO) or through cell-to-cell contact. In an environment composed by strong inflammatory components, MSCs have the ability to change the T-helper 1 (Th1) proinflammatory profile into a T-helper 2 (Th2) anti-inflammatory profile [23, 38].

The effects of MSCs on B cells remains contradictory although there is evidence that MSCs have close interactions with B cells. MSCs are able to inhibit B cell proliferation through cell-to-cell contact and through an arrest in the G0/G1 phase of the cell cycle [45]. Moreover, MSCs suppress plasma cell differentiation induced by allostimulation and immunoglobulin (Ig) production. Studies have also suggested that, although MSCs are able to suppress B cells which are activated by several stimuli, they are incapable of modulating naive or memory B cells [14, 23, 38, 46].

Concluding, MSCs regulate immune responses such as altering antibody production by B-lymphocytes, shifting T-lymphocyte subtypes, and inducing immune tolerance to allogeneic transplants due to the lack of MHC-II expression and co-stimulatory molecules such as CD40, CD80 and CD86, since they escape the recognition and action of T cells and NK receptors [6, 35]. However, their immunomodulatory capacity is not yet fully understood and there are still variable results concerning immunomodulatory therapies with MSCs [15]. A deeper understanding of the mechanism through which the MSCs deriving from veterinary species modulate inflammation and contribute to the healing process, will beneficiate human beings as well as animals [23].

## Therapeutic strategies with mesenchymal stem cells in immune-mediated disorders

### Canine atopic dermatitis

Atopic dermatitis (AD) is the most frequent dermatopathy in dogs and shares many characteristics with the human disease [4, 19]. This chronic multifactorial disorder is associated with breed predilections, polymorphisms at specific gene loci, altered gene expression and specific allergens [47–49]. It is characterized by a dysfunction of the skin barrier due to changes in lipid (ceramides) and protein (filaggrin) composition, modifications in the stratum corneum and loss of water from the transepidermis, which predisposes to a higher allergen penetration [4, 47–49]. The skin of atopic dogs tends to produce less antimicrobial agents (defensins, cathelicidins, etc.) usually leading to secondary infection. Infectious agents such as *Staphylococcus pseudointermedius*, *Staphylococcus intermedius* and *Malassezia pachydermatis* worsen the clinical presentation of AD (pyoderma and otitis) and induce a phenomena of allergic sensitisation with large amounts of lgE antibodies [4, 50]. When allergenic load overcomes a certain threshold, mast cells will activate leading to consequent allergic response and its clinical presentation.

According to the 2015 updated guidelines from the International Committee on Allergic Diseases of Animals (ICADA), the treatment of acute and chronic AD is based on three key points, as described in Table 2 [51].

**Table 2:** 2015 updated guidelines of acute and chronic atopic dermatitis treatment [51]

	Acute AD	Chronic AD
Identification and avoidance of flare factors	Elimination of allergenic flare factors (fleas, food and environmental); Evaluation of the use of antimicrobial therapy if clinical signs of infection with bacteria or yeast are present on the skin or in the ears.	Dietary restriction-provocation trials in dogs with nonseason signs; Implementation of an effective flea control regimen; Performance of allergen-specific intradermal and/or IgE serological tests to identify possible environmental allergen flare factors; Implementation of house dust mice or other allergen control measures; Evaluation of the use of antimicrobial therapy (terbinafine or itraconazole once a day [SID] for two consecutive days each week for 3 weeks to treat flares provoked or exacerbated by *Malassezia* skin infections).
Improvement in skin and coat hygiene and care	Bathing with a non-irritating shampoo containing lipids, complex sugars and antiseptics or phytosphingosine, raspberry oil and lipids.	Bathing at least once weekly with a non-irritating shampoo or an antiseborrheic/ antimicrobial shampoo and dietary supplementation with essential fatty acids.
Reduction of pruritus and skin lesions	Topical glucocorticoids sprays for localized lesions; Oral glucocorticoids (prednisolone, prednisone or methylprednisolone given at 0.5 to 1.0 mg/kg per day SID or two times a day [BID]) or oral oclacitinib (0.4 to 0.6 mg/kg BID for up to 14 days) for widespread or severe lesions.	Topical glucocorticoids sprays for localized lesions; Oral glucocorticoids (prednisolone, prednisone or methylprednisolone given at 0.5 mg/kg SID or BID), oral cyclosporine (5 mg/kg SID until satisfactory control of clinical signs), oclacitinib (0.4 to 0.6 mg/kg BID for 14 days and then SID) or injectable interferons (recombinant canine interferon-gamma given subcutaneously [SC] at 5.000–10.000 units/kg three times weekly for four weeks and then once weekly) for widespread or severe lesions. These drugs should not be combined together in the long term to reduce the risk of immunosuppression.

With the aim of preventing the reappearance of clinical signs, some strategies can be developed, such as avoidance of known flare factors, consideration of proactive intermittent topical glucocorticoid therapy and implementation of allergen-specific immunotherapy, if feasible [51].

Some adverse effects are seen with these treatments, especially with its long-term use. Unfortunately, due to AD pathophysiology, glucocorticoids are frequently needed. Systemic administration of these drugs might result in polyuria, polydipsia, polyphagia, changes of behaviour (including aggressiveness) and, depending on the initial dose, iatrogenic hyperadrenocorticism [51].

Another therapy with a canonized anti-canine IL-31 monoclonal antibody - lokivetmab (ZTS-00103289) - has recently demonstrated efficacy in reducing pruritus in canine atopic dermatitis trials [52, 53]. This monoclonal antibody is administrated subcutaneously in the animal and binds specifically to circulating IL-31, thereby inhibiting its binding to the IL-31 receptor [52, 53]. However, its effects are still poorly understood.

Over the last few years, the immunomodulatory effect of MSCs therapy has been described in animal models and in human beings, showing a significant improvement in the clinical presentation by inhibiting the activation of T and B cells and consequent release of anti-inflammatory cytokines (IL-10, TGF-β), by decreasing the proliferation of IL-4 and IFN, and by decreasing the production of lgE [4].

Hall et al. (2010) carried out a clinical trial with five AD canine patients (Table 3). All the patients were treated with a single dose of autologous adipose stem cells (ASCs). The dosage of intravenous (IV) 1 × 10^6^ cells (1.3 million cells/kg) applied in this trial was substantially lower than the dosage applied in other trials and lower than the dosages usually applied in human trials (≥2 × 10^6^/kg of body weight). Although the injections had been considered safe, no signs of progress were observed during this trial with the ASCs treatment [54].

**Table 3:** Clinical trial carried out with mesenchymal stem cells in canine atopic dermatitis

Specie (number)	Treatment	Results	Conclusion	Reference
Dog (5)	Single IV autologous ASCs (1.3 million cells/kg)	No benefits of ASCs treatment were observed.	The dosage of ASCs was lower than employed in other studies. The results are inconclusive.	[54]

Jiménez & Guerrero (2017) have also published in the Clindervet journal a study on the use of MSCs in veterinary dermatology, where they report a successful case of canine AD with MSCs-based treatment. They obtained significant improvement in clinical signs with no complications mentioned [4].

### Feline chronic Gingivostomatitis

Feline chronic gingivostomatitis (FCGS) is a severe, idiopathic, inflammatory oral disease characterized by severe inflammation of the gingiva, buccal mucosa and caudal oral mucosa, that affects approximately 0.7–10% of the general cat population [55–57]. The aetiology of FCGS is poorly understood, nonetheless it has been suggested that microbial factors and alterations in the innate immune response may play an important role in the pathogenesis of this disorder [58]. Researchers believe that the factor contributing the most to the development of this disorder is the presence of bacterial plaque and that the development of FCGS is due to an immune abnormality, specifically related to the inflammatory mediators produced by lymphocytes and plasma cells in response to infectious agents (e.g. feline calicivirus [FCV], feline leucemia [FeLV], feline herpesvirus, feline immunodeficiency virus [FIV] and *Bartonella henselae*). However, these infectious agents may not be responsible for the disease, and rather be mere contributors to the patient’s morbidity in the healing phase of the treatment [57, 59]. Histologically, the injuries in cats are characterized by an inflammation with lymphocytes, mostly effector T cells and B cells [22].

This disorder causes painful mucosal lesions that markedly reduce the quality of life. Clinical signs vary from pain and moderate to severe oral discomfort, inappetence, loss of weight, reduced grooming and ptyalism [21].

According to the Portuguese Association of Veterinarians Specialized in Companion Animals (APMVEAC),^1^ so far there is no treatment against FCGS that is 100% effective, and this may result in euthanasia of several affected cats. Approximately 70% of cats respond to standard treatment consisting of total or partial teeth extraction. The remaining 30% do not react to teeth extraction and require therapy with antibiotics, corticosteroids and other pain relief medication throughout their lives [21, 55].

A successful treatment requires the minimization of oral bacteria (Table 4), therefore the therapeutic plan should be started with the improvement of the animal’s oral hygiene with professional veterinary dental cleaning with follow-up controls [57, 60].

**Table 4:** Conventional and immunomodulatory therapeutic approach to feline chronic gingivostomatitis

	Feline Chronic Gingivostomatitis
Teeth extraction	All teeth with inflammation of the gingiva and adjacent mucosa should be removed, as well as teeth with dental resorption lesions and with advanced periodontal disease [21, 55].
Oral hygiene	Professional veterinary dental cleaning with follow-up controls [57, 60].
Corticosteroids	Prednisolone after tooth extraction (3–4 mg/kg SID during 3 to 4 weeks) [57, 59]. Although its use is still controversial among authors.
Cyclosporine	Cyclosporine is a potent immunossupressive that minimizes IL-2 expression and subsequently minimizes T cell numbers. Usually microemulsified cyclosporine suspension (2–5 mg/kg PO BID) is used. However a modified cyclosporine has recently been introduced (7.5–10 mg/kg PO SID) and needs to be administrated in higher dosages to attain proper blood levels [57, 59, 61].
Feline recombinant interferon omega	This drug has not displayed adverse effects and is licensed to treat retroviral infections. Studies have shown that interferon delivered transmucosally was as effective as prednisolone in decreasing clinical signs [57, 59].
CO2 laser therapy	The purpose of this therapy is to carbonize inflamed tissue, resulting in the formation of scar tissue. This scar tissue is considerably less likely to become inflamed over time. This therapy may be repeated in 4 to 6 weeks, if needed [57, 59, 62].

All teeth with inflammation of the gingiva and adjacent mucosa should be removed, as well as teeth with dental resorption lesions and with advanced periodontal disease. Tooth extraction can be multiple or radical depending on affected teeth, and should begin with the removal of the premolar and molar teeth when affected. If incisive and canine mucous membrane is inflamed, these teeth should also be removed, leading to a radical extraction with removal of all teeth (Table 4). The transition to canned pet food (with an appetite stimulant if necessary) before the surgery is an important step to minimize mouth pain [57]. For the surgical approach, pain control with buprenorphine (0.02 mg/kg sublingually three times a day [TID] or BID) and gabapentin (5–10 mg/kg BID or SID) is recommended, and a nonsteroidal anti-inflammatory (NSAID) might also be applied (e.g. robenacoxib 1–2 mg/kg orally [PO] SID), during post-surgery. The use of systemic antibiotics instead of partial or total teeth extractions is unwise and only contributes to the patient’s likely resistance to antibiotics [57].

The authors define gingivostomatitis refractory to dental extraction when there is no improvement of clinical signs up to 60 days after teeth removal, which comes as a considerable therapeutic challenge for the physician. In refractory situations treatment with anti-inflammatory or immunomodulatory therapies might be considered (Table 4) [57, 59, 62].

ASCs therapy is another option increasingly used. The ability of MSCs to inhibit T-cell proliferation and induce T-cell anergy suggests that therapy with MSCs can be quite promising for the treatment of FCGS. Arzi et al. (2016) carried out a clinical trial with seven FCGS patients, non-responsive to radical teeth extraction and immunosuppressive therapies. Treatment was based on two IV administrations of 2 × 10^7^ autologous ASCs (~ 5 million cells/kg) with 3 to 4 weeks apart whose results are displayed on Table 5. The authors applied flow cytometry to compare CD8 expression with treatment reaction. It was found that cats with < 15% CD8 T cytotoxic cells (with low expression of those cells) were 100% responsive to therapy, while cats with > 15% did not react to treatment. Relative absence of CD8 cells may be a biomarker to predict the response to therapy using adipose stem cells [55].

**Table 5:** Clinical trials carried out with mesenchymal stem cells in feline chronic gingivostomatitis

Specie (number)	Treatment	Results	Conclusion	Reference
Cat (7)	Two IV autologous ASCs (~ 5 million cells/kg)	Complete remission (3 cats), substantial improvement (2 cats), no response (2 cats)	Autologous therapy may be slightly more effective	[55]
Cat (7)	Two IV allogeneic ASCs (~ 5 million cells/kg)	Complete remission (2 cats), substantial improvement (2 cats), no response (3 cats)		[21]

In 2017 Arzi et al. carried out a similar trial using the same dosage and time spans in seven cats with FCGS, yet the therapy applied used allogeneic ASCs (results are displayed on Table 5). These results suggest that autologous therapy may be somewhat more effective, particularly in severely affected cats, and may cause improvement or complete remission of signs more rapidly than with allogeneic therapy [21].

### Inflammatory bowel disease

Inflammatory bowel disease (IBD) is a chronic inflammatory enteropathy characterized by intestinal inflammation and persistent or frequent gastrointestinal signs, that do not respond to food trials or antimicrobial treatments, therefore requiring immunosuppressive treatment [63–65]. The aetiology of IBD in veterinary medicine is not fully understood, although there are similarities with human IBD (Crohn’s disease and ulcerative colitis) [66]. Idiopathic IBD is the most common aetiology in dogs and cats possibly resulting from the breakdown of immunologic tolerance to luminal antigens (commensal bacteria and dietary components), most likely due to disruption of the mucosal barrier, dysregulation of the immune system, or disturbances in the microbiome, with upregulation of Toll-like Receptors (TLRs) [20, 22, 63, 67]. Genetic factors are likely to contribute to the pathogenesis of IBD [63].

Currently, there are no definitive guidelines for the treatment of IBD so it can undergo individual variability according to the patient’s record (Table 6). The proposed treatment aims to reduce gastrointestinal signs (e.g. vomit and diarrhoea), increase appetite and weight, and reduce intestinal inflammation [63].

**Table 6:** Conventional and immunomodulatory therapeutic approach to inflammatory bowel disease

	Inflammatory Bowel Disease
First line approach	Sequential treatment trials of parasiticides, an exclusion diet, and antibacterials to exclude known causes of inflammation before the immunosuppressive treatment [63].
Corticosteroids	Oral prednisolone (1 mg/kg BID) [63, 64, 68].
Other immunosuppressive agents	Azathioprine (frequently used in dogs when IBD cannot be effectively managed with glucocorticoids) or cyclosporine (inhibits the production of IL-2) [63, 64, 68].

A dietary modification with limited antigen, highly digestible and with a single source of protein has been recommended. These exclusion diets may also help resolve any secondary sensitivities to dietary components that might have arisen after disruption of the mucosal barrier [63, 68].

Treatment with antimicrobials can be justified in IBD, partially to treat any secondary Small Intestinal Bacterial Overgrowth (SIBO) and partially because of the importance of bacterial antigens in the pathogenesis of IBD [63, 68]. Metronidazole is the preferred drug for small animals [63].

Immunosuppressive medication with corticosteroids is the treatment of choice in most cases, although cushingoid side effects are common but transient as the dosage is reduced. Azathioprine is an alternative drug that has good steroid-sparing properties and is frequently used in dogs when IBD cannot be effectively managed with glucocorticoids or when the glucocorticoid dose has to be reduced. If there is still a poor response, cyclosporine is another alternative agent, because it inhibits the production of IL-2. However its efficacy has not yet been fully validated [63, 64, 68].

The animal owners’ decision to carry out a treatment for this chronic disease might be a concern because not every animal responds to treatment. For this reason, alternative approaches became necessary [22] Applying MSCs as an alternative treatment for IBD is still a very recent conception. However, the use of this therapy in clinical trials on human beings with inflammatory gastrointestinal and immune disorder has proven to be effective and safe [20, 67].

Pérez-Merino et al. (2015) [67] carried out a clinical trial with eleven dogs with IBD that had received standard treatment (elimination diet, corticosteroids, antibiotics, antidiarrhoeal and antiparasitic drugs) but did not achieved a satisfactory response (Table 7). These animals went through a period of washout of at least 3 weeks before the trial was undertaken. All dogs received a clinical score using the Clinical Inflammatory Bowel Disease Activity Index (CIBDAI) and the Canine Chronic Enteropathy Clinical Activity Index (CCECAI) scoring system. Every dog was treated with a single ASCs IV infusion (2 × 10^6^ cells/kg bodyweight). After 2 weeks of ASCs therapy, a clinical response occurred in all dogs, but clinical remission (defined by a reduction of initial CIBDAI and CCECAI > 75%) occurred in 9/11 dogs at day 42. The remaining two dogs showed a partial response with an initial reduction of 69.2 and 71.4% in CIBDAI and CCECAI respectively. In conclusion, the administration of a single IV infusion of allogeneic MSCs was well tolerated by the patients and seemed to produce clinical benefits in dogs with severe IBD.

**Table 7:** Clinical trials carried out with mesenchymal stem cells in canine and feline inflammatory bowel disease

Specie (number)	Treatment	Results	Conclusion	Reference
Dog (11)	Single IV allogeneic ASCs (2 × 10^6^ cells/kg)	After 2 weeks of MSCs therapy, a clinical response occurred in all dogs	ASCs was well tolerated and appeared to produce clinical benefits in dogs and cats with IBD	[67]
Cat (7)	Two IV allogeneic ASCs (2 × 10^6^ cells/kg)	Improved clinical signs in 5/7 MSCs-treated cats		[69]

Another study using MSCs IV therapy in spontaneous feline enteropathy showed a safe and positive clinical response [69] as described in Table 7. Seven cats with diarrhoea for at least 3 months, received two IV injections of 2 × 10^6^ cells/kg from cryopreserved feline ASCs, while four cats with a similar clinical condition received saline placebo. Improvement of clinical signs was observed in 5/7 cats that were treated with stem cells after 1 to 2 months, unlike the placebo group which did not display any progress. With this trial it is possible to conclude that MSCs therapy was well tolerated and potentially effective in the treatment of feline chronic enteropathy, although these preliminary results require significant follow-up study.

### Feline asthma

Asthma is a common lower airway inflammatory disease (LAD) in cats that is associated with substantial morbidity and occasional mortality [70, 71]. The word *asthma* suggests reversible bronchoconstriction and a prevailingly eosinophilic inflammation of the airways and primary symptoms include cough, wheeze and respiratory distress [72, 73].

The main factors responsible for triggering asthma are extensive and complex and they include infectious, environmental, allergic and genetic elements [74]. In cats there is evidence that asthma is mediated by an allergic response after exposure to inhaled aeroallergens. These aeroallergens induce stimulation of a Th2 response and lead to production of a variety of cytokines that trigger molecular switches leading to pathologic changes in airways [70, 72].

Currently there is no curative treatment for feline asthma. Treatment goals consist of reducing airway inflammation, reducing airway hyper reactivity and bronchoconstriction (which relieves airflow limitation), ameliorating airway remodelling and removing the underlying cause, if known [73]. Treatment of acute dyspnea associated with LAD in cats starts with oxygen supplementation and minimal handling/stress reduction. The conventional therapeutic approaches for acute and chronic asthma, described in Table 8, are based on glucocorticoids (which are the gold standard of therapy for reducing airway inflammation) and bronchodilators [70, 73].

**Table 8:** Conventional and immunomodulatory therapeutic approach to feline asthma

	Acute Asthma	Chronic Asthma
Glucocorticoids	Dexamethasone (0.15–1 mg/kg intramuscular [IM] or IV) is indicated in cats that show signs of acute dyspnea [70, 73].	Oral prednisolone (0.5–1 mg/kg BID) is recommended for the first 7 to 14 days. Once clinical signs are well controlled, the dose can be gradually reduced over 2 to 3 months to once a day. Inhaled fluticasone (110 mcg BID for 2 to 3 weeks) is an alternative although it is not useful in a crisis because it takes about 10 to 14 days to become effective and, since pets cannot be trained to inhale correctly, administration of aerosolized drugs requires the use of mask. Injectable methylprednisolone acetate (10–20 mg/cat IM or SC every 4 to 12 weeks) may also be used [70, 73].
Bronchodilators	β2-receptor agonists, such as terbutaline (0.01 mg/kg IM or SC), or albuterol (90 mcg inhaled) to reduce bronchoconstriction and relieve airflow limitation [70, 73].	The most generally used are β2-receptor agonists, namely terbutaline (0.1–0.2 mg/kg PO TID or BID), and less commonly methilxanthine derivates such as theophylline (the recommended dose of sustained-release theophylline in cats is 20 to 25 mg/kg PO SID, and for non-sustained-release theophylline 4 mg/kg TID or BID) [70, 73].
Allergenic-specific immunotherapy	—	Intravenous or subcutaneous allergenic-specific immunotherapy was proved to decrease eosinophilia airway inflammation and is generally associated with minimal side effects [70, 75].
Inhibitors of tyrosine kinase	—	Inhibitors of tyrosine kinase are small molecules that block ATP-binding site of kinases. During a trial model of feline asthma, this therapy has proven to be efficient in reducing airway inflammation [68, 76].
Cyclosporine	—	Cyclosporine inhibits T-cell activation and blocks the development of a Th2 phenotype and the associated Th2-eosinophil interactions. In a feline asthma experimental study, Mitchell et al. (1998) [77] demonstrated that cyclosporine did not inhibit the early phase response to allergen challenge, but it was effective at reducing airway hyperresponsiveness to acetylcholine and airway remodeling [78].

Although conventional therapy reduces inflammation and dyspnea in a considerable number of cats, there is still no therapy capable of preventing or reversing all pathological aspects of asthma. Some cats continue resistant to this therapy with persistent clinical signs and eosinophilia in the airways. Other cats are affected by concurrent diseases (e.g. cardiac disease or diabetes mellitus) where the use of glucocorticoids is not advised. This is the reason why we need new therapies for the treatment of asthma [70, 71] namely allergenic-specific immunotherapy, inhibitors of tyrosine kinase, cyclosporine and MSCs therapy (Table 8).

Therapy with MSCs would be ideal given their immunomodulatory abilities (they modulate Th2 lymphocyte activity) and their capability to pass through the lung when administered intravenously [22]. Murine asthma models have demonstrated that stem cells can reduce airway eosinophilia, airway hyper-responsiveness and promote airway remodelling [70]. Two pilot trials carried out in cats have been published to test the efficiency of MSCs therapy. Both trials involved sensitization to Bermuda grass allergen, which resulted in the development of an asthmatic phenotype with airway eosinophilia and airway hyper-responsiveness.

The first study involved six cats with acute asthma, 4/6 received five intravenous infusions of allogeneic MSCs (infusions varied between 2 × 10^6^ and 1 × 10^7^ cryopreserved MSCs per cat) and 2/6 received a saline placebo (Table 9). Cats treated with MSCs achieved a decrease in airway eosinophilia and diminished airway hyper-responsiveness at day 133 when compared to the group that was exclusively administrated with placebo. In this trial, lung attenuation and bronchial wall thickness were also assessed by computerized tomography, and it was verified that the score of these parameters was substantially reduced in cats treated with MSCs 9 months later [71].

**Table 9:** Clinical trials carried out with mesenchymal stem cells in feline asthma

Species (number)	Treatment	Results	Conclusion	Reference
Cat (6) Acute asthma	Five IV allogeneic ASCs (varied between 2 × 10^6^ and 1 x 10^7^per cat)	Decreased in: airway eosinophilia, hyper-responsiveness and airway remodeling	ASCs therapy had a positive effect on airway remodeling	[71]
Cat (9) Chronic asthma	Six IV allogeneic ASCs (with range amplitude of 0.36–2.5 × 10^7^ MSCs/infusion)	Decreased in airway remodeling		[79]

The second study involved nine cats with chronic asthma, 5/9 received six intravenous infusions of allogeneic MSCs (with range amplitude of 0.36–2.5 × 10^7^ MSCs/infusion) and 4/9 received a saline placebo (Table 9). Unlike the previous trial, cats suffering from chronic asthma that were treated with MSCs did not experience a decrease in airway eosinophilia and diminished airway hyper-responsiveness compared with placebo group. However, there was a significant reduction in the lung attenuation and in the bronchial wall thickness observed through computerized tomography 8 months after treatment, once again indicating a positive effect on airway remodelling [79].

Therapy with ASCs proved to have a positive effect on remodelling airways in the two pilot trials controlled by placebo and using feline asthma models. Moreover, the results attained from this cell therapy were more favourable when treatment was carried out in an acute stage of the asthma.

## Current status and future prospects

The multipotent and non-teratogenic properties of MSCs led to the conclusion that these cells could be clinically used to regenerate injured tissues and to treat immune-mediated disorders due to their immunomodulatory potential. Over the last few years some researchers have questioned the efficiency of the treatment with stem cells. There is sparse evidence suggesting that the primary function of MSCs is its differentiation in in vivo new tissues, questioning the importance of differentiation to the therapeutic properties of such cells when injected in a naive state [13, 82].

There are still several questions concerning the use of MSCs: their immunomodulation mechanism has not yet been completely understood and the combined results of this therapy require a better scientific clarification. The use of allogeneic vs. autologous MSCs therapy might contribute to the differences in efficacy observed in some clinical trials. On the other hand, the in vitro expansion of MSCs before clinical usage might take weeks before it provides enough cells to administrate with therapeutic effect, resulting in loss of stemness [82].

In fact, different routes of administration result in different risks for the patient. Systemic administration can lead to the entrapment of MSCs in the microvasculature or lung, causing dangerous side effects for the patient, such as pulmonary emboli [12]. Also, these cells, once administered, are almost 90% lost because of physical stress, inflammation, hypoxia, or immunogenic rejection. To reach therapeutic efficacy, a large number of cells may be required, increasing the potential risk of teratoma formation. Therefore, new studies should be carried out in order to define the minimum therapeutic dose of MSCs [12].

In order to overcome these obstacles to treatment, careful evaluation of appropriate cell sources, good quality control systems, standardized protocols for cell culture and their differentiation, expansion and cryopreservation are necessary. Therefore, the regulation mechanism of MSCs to produce soluble factors and the way these factors are capable of modulating cells of the immune system are key questions that underlie the successful immunomodulation effects of MSCs. All these factors combined with genetically modified MSCs might open a way for the development of an effective cell therapy for multiple animal and human immune disorders.

Despite current contradiction found among researchers concerning the efficacy of MSCs, this revision has demonstrated a generally positive result of the use of these cells in the treatment of immune-mediated disorders in veterinary medicine. Even so, MSCs-based therapy is currently requiring, more than ever before, a complete analysis and reconsideration in the hope to overcome its limitations in coming trials. In the future, this approach could be more cost-effective than actual conventional treatments due to the recent development and continuous success regarding MSCs application in immune-mediated diseases.

## Conclusions

MSCs immunomodulatory properties make them a unique cell type capable of repairing tissue and organ injuries caused by chronic inflammation or autoimmune disorders.

According to the results of aforementioned trials, it is possible to verify a positive balance in the use of MSCs for the treatment of FCGS, IBD and feline asthma. Concerning FCGS and IBD, one or two infusions of stem cells were sufficient to attain considerably favourable results, as most of the animals involved experienced improvements of the clinical signs and none of them revealed any adverse effects.

In feline asthma, the clinical trials required a higher number of stem cells infusions (five to six) to provide for a proper passage of these cells towards the lung, and the results were equally satisfactory. Furthermore, the results achieved from this cell therapy were more favourable when treatment was carried out with autologous MSCs and in acute asthma.

Canine atopic dermatitis has not displayed results as satisfactory as the other observed diseases. However, only one clinical trial has been carried out in a sample of five dogs, which included a sole IV administration of MSCs at a lower dosage than the dose used in other similar trials. This might be one of the reasons for the failure of the therapy. Meanwhile, there are several unpublished studies that claim success in treating AD by injecting MSCs both IV and IM on the main areas affected by the disease. Despite this claim, these results still lack validation and standardization of the total number of cells injected, as different studies report using different numbers of cells per kg of body weight.

**Fig. 5:**
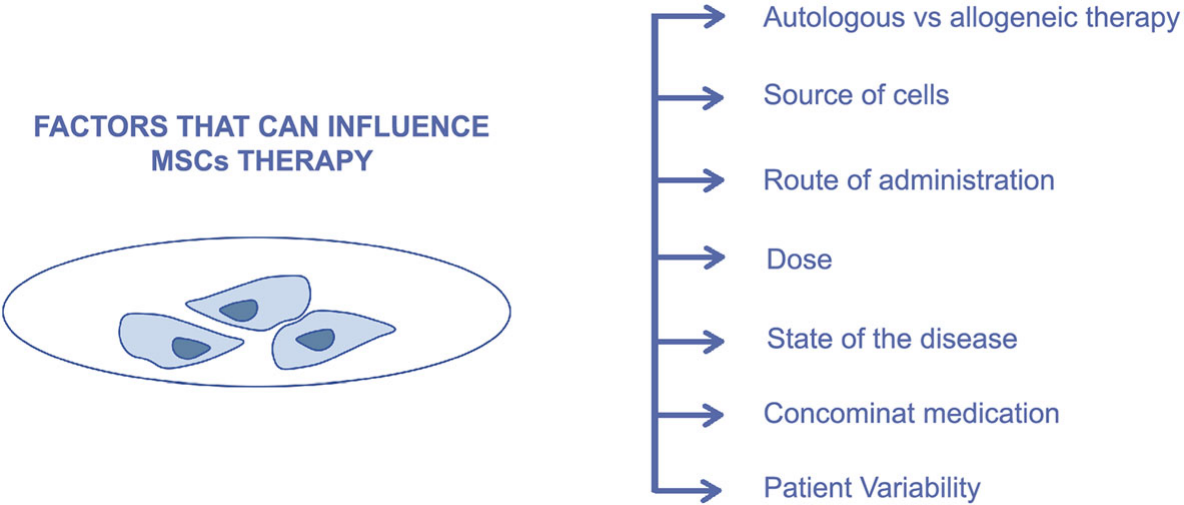
Factors that can influence mesenchymal stem cells therapy

Although MSCs can bring a promising future to the treatment of the majority of these disorders, the considerable variability of their quality derived from different donors (autologous vs allogeneic therapy), different tissues, different administration routes, different dosages, individual variability of each patient (e.g. age of the patient) and state of the disease, might limit its therapeutic benefit (Fig. 5).

Canine atopic dermatitis, feline chronic gingivostomatitis, inflammatory bowel disease and feline asthma are relatively common diseases among veterinary medicine, which is why they were widely considered throughout this revision. Nevertheless, the same rational used in these disorders may be applied to several others with a similar immunomediated nature, namely systemic lupus erythematosus, pemphigus foliaceus, perianal fistula and immune mediated cutaneous vasculitis [4, 80, 81]. All these pathologies are characterized by a dysfunction of the immune system with a pathologic response mediated by Th1 cells and inflammatory mediators. Since MSCs are able to suppress the proliferation of T cells and modulate their response (change the Th1 proinflammatory profile into a Th2 anti-inflammatory profile) through the secretion of several soluble factors or through cell-to-cell contact [38], they will certainly become an important therapeutic approach to treat any of these pathologies. Nevertheless, sufficient clinical trials have not yet been performed to confirm the success and validity of the therapy.

The main obstacle to MSCs therapy is individual and species diversity, and also the inconsistency of protocols applied to date, since there are still very few clinical trials performed in veterinary medicine and most of them have used small samples. Both physicians with expertise in this field and regulatory agencies, need to work together towards the standardization and quality assurance of cellular therapies being applied clinically. The way we practice medicine is changing and evolving rapidly, and although it is difficult to predict where we will be in the near future, cellular therapies certainly seem to have come to stay.

## Data Availability

All data generated or analysed during this study are included in this published article [and its additional files].

## Abbreviations

AD: Atopic Dermatitis
APMVEAC: Associação Portuguesa de Médicos Veterinários Especialistas em Animais de Companhia
ASC: Adipose-derived Stem Cell
ATP: Adenosine Triphosphate
BID: Bis In Die (In Latin, two times a day)
BM: Bone Marrow
BMSC: Bone Marrow-derived Stem Cell
CCECAI: Canine Chronic Enteropathy Clinical Activity Index
CD: Cluster Differentiation
CIBDAI: Clinical Inflammatory Bowel Disease Activity Index
DCs: Dendritic Cells
ESCs: Embryonic Stem Cells
FCGS: Feline Chronic Gingivostomatitis
FCV: Feline Calicivirus
FDA: Food and Drug Administration
FeLV: Feline Leukemia Virus
FIV: Feline Immunodeficiency Virus
HGF: Hepatocyte Growth Factor
HO: Hemoxygenase
IBD: Inflammatory Bowel Disease
ICADA: International Committee on Allergic Diseases of Animals
IDO: Indolamine 2,3-dioxygenase
IFN-γ: Interferon gamma
Ig: Immunoglobulin
IL: Interleukin
IM: Intramuscular
iPSCs: Induced Pluripotent Stem Cells
IV: Intravenous
kg: Kilogram
LAD: Lower Airway Disease
mg: Miligram
mgc: Microgram
MHC-II: Major Histocompability Complex II
MSCs: Mesenchymal Stem Cells
NG2: Neural/Glial antigen 2
NK: Natural Killer
NO: Nitric Oxide
NSAID: Nonsteroidal Anti-Inflammatory Drug
PDGF-Rβ: Beta-type Platelet-Derived Growth Factor Receptor
PGE_2_: Prostaglandin E_2_
PO: Per os (In Latin, orally)
SC: Subcutaneous
SIBO: Small Intestinal Bacterial Overgrowth
SID: Sem’el In Die (In Latin, once a day)
TGF-β: Transforming Growth Factor β
Th1: T helper 1
Th2: T helper 2
TID: Ter In Die (In Latin, three times a day)
TLRs: Toll-like Receptors
TNF: Tumor Necrosis Factor

## Footnotes

1 Associação Portuguesa de Médicos Veterinários Especialistas em Animais de Companhia (APMVEAC)
